# Quality variation and biosynthesis of anti-inflammatory compounds for *Capparis spinosa* based on the metabolome and transcriptome analysis

**DOI:** 10.3389/fpls.2023.1224073

**Published:** 2023-07-17

**Authors:** Xiaoying Liu, Alimu Aimaier, Weilan Wang, Yuliang Dong, Peng Han, Jiang He, Lihong Mu, Xinhui Wang, Jinyao Li

**Affiliations:** ^1^ Xinjiang Key Laboratory of Biological Resources and Genetic Engineering, College of Life Science and Technology, Xinjiang University, Urumqi, China; ^2^ Key Laboratory of Uygur Medicine, Xinjiang Institute of Materia Medica, Urumqi, China; ^3^ College of Ecology and Environment, Xinjiang University, Urumqi, China

**Keywords:** *Capparis spinosa*, quality variation, anti-inflammation, metabolome, RNA-seq

## Abstract

**Introduction:**

*Capparis spinosa* L. fruits as edible and medicinal plant, has anti-inflammatory activities. The different morphological characteristics of *C. spinosa* fruits from Ili, Turpan, and Karamay may affect their anti-inflammatory components and functions.

**Methods:**

The anti-inflammatory activity of *C. spinosa* fruit was assessed using an LPS-induced inflammatory cell model. Furthermore, the differences in anti-inflammatory compounds were analyzed by metabolome and RNA-seq. Additionally, the anti-inflammatory mechanism was elucidated using network pharmacology.

**Results:**

In the study, we found that the 95% ethanol extracts (CSE) obtained from the three kinds of fruits showed remarkable anti-inflammatory effects both *in vivo* and *in vitro*. However, the CSE derived from Ili fruits significantly reduced CD86 levels on DCs. As a result of metabolomic analysis, the metabolic profiles of Ili fruits differed significantly from those of the other two habitats, which were consistent with transcriptome analysis. A total of 15 compounds exhibiting anti-inflammatory activity were subjected to screening, revealing a greater accumulation of flavonoids in the Turpan and Karamay districts. Notably, phenolic compounds were identified as the principal anti-inflammatory components in *C. spinosa*.

**Conclusion:**

There were significant differences in the morphology, metabolites, transcriptional levels, and anti-inflammatory activity of *C. spinosa* from the three districts.

## 1 Highlights

1. The three origins of *Capparis spinosa* L. fruits have anti-inflammatory effects, but the anti-inflammatory effects of *Capparis spinosa* L. vary by origin.
2. Phenolic compounds are the main anti-inflammatory substances in *Capparis spinosa* L. fruits.
3. A gene that increases ferulic acid synthesis was identified.

## 2 Introduction

*Capparis spinosa L.* (English name: Caper; Chinese name: Cishangan) belongs to the Capparidaceae family, Capparis genus. This shrub is distributed throughout the Mediterranean basin and is particularly common in the northwest region of China (Cao et al., 2010). It is also known as caper, wild watermelon in China. *C. spinosa* is extremely drought-resistant and can be found in semi-desert and desert regions as well as in gobi, sandy and gravelly slopes at low elevations (Khatib et al., 2016). *C. spinosa* is mainly distributed in the Turpan, Junggar, Tarim basins, and Ili River valleys of Xinjiang, China (Editorial Committee of Flora of Xinjiang, 1995; Editorial Committee of Flora of China and Chinese Academy of Sciences, 1999).

*C. spinosa* fruits and flower buds are fermented and consumed as foods or condiments (Francesca et al., 2016). Fruits have also traditionally been used for pharmacological purposes, especially for treating inflammation and arthritis through external applications. Studies showed that capers also have antidiabetic (Jalali et al., 2016), anti-hyperlipidemic (Khavasi et al., 2017), antiallergic (Trombetta et al., 2005), anti-oxidation (Tlili et al., 2015), anti-tumor (Ji and Yu, 2015), hepatoprotective (Kalantari et al., 2018) and neuroprotective effects (Mohebali et al., 2016; Rahimi et al., 2020). *C. spinosa* contains many kinds of biochemical compounds, including flavonoids, alkaloids, terpenoids, polyphenols, lipids, essential oils, and glycosides (Zhang et al., 2018).

Intraspecific variation is obvious, so traditional classification methods are difficult to accurately identify the taxonomic position of polymorphic *C. spinosa* (Fici, 2001; El Zayat et al., 2020). A genetic study based on AFLP fingerprinting confirmed this phenomenon (Inocencio et al., 2005). Our observation showed that the capers from Ili were significantly different from those from Turpan in terms of morphology. Meanwhile, through our investigation, we found that more than 60% of the capers in Xinjiang came from Turpan. It has not been studied whether such morphological and habitat differences affect the anti-inflammatory components and activity of *C. spinosa*.

The variation of habitat and morphology of traditional Chinese medicine (TCM) sometimes affects the content of active substances. The dry root and rhizome of *Salvia miltiorrhiza* Bunge (Danshen) are a TCM. Tanshinones are important active compounds, and their accumulation in the pericardium affects root color. The contents of Tanshinone IIA and Tanshinone I in the orange roots of the mutant were significantly reduced. However, the key enzyme genes involved in biosynthesis did not differ at the transcriptional level between the two kinds of *S*. *miltiorrhiza* (Zhan et al., 2019). *Cistanche deserticola* is an edible and medicinal plant, with phenylethanoid glycosides (PhGs) as its major active compounds. However, the content of PhGs in samples from three ecotypes grown in saline-alkali land, grassland, and sandy land was significantly different. Compared with other ecotypes, the content of PhGs was higher in saline-alkali soils, which may be due to the up-regulation of PhGs biosynthesis genes (Sun et al., 2020). Sixty samples of *glycyrrhiza uralensis* from different districts in Gansu, China, were analyzed, and the results showed significant differences in active compounds (Bai et al., 2020).

In this study, we conducted a comparative analysis of the anti-inflammatory activity, metabolites, and transcriptome data of *C. spinosa* fruits from different habitats. We identified the habitats with the strongest anti-inflammatory activity and screened the major anti-inflammatory components in *C. spinosa* fruits. Through gene-metabolic network analysis, we identified several key metabolic pathways and genes that influence the synthesis of anti-inflammatory compounds. These findings provided new insights into the further development, cultivation, and genetic modification of *C. spinosa*.

## 3 Results

### 3.1 Fruit morphology and sampling location information

**Figure 1:**
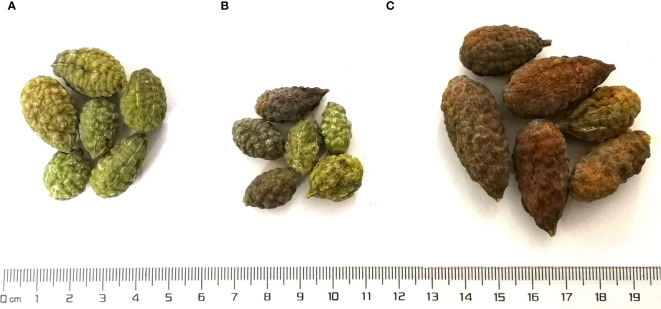
The fruits from three districts. **(A)** Turpan; **(B)** Karamay; **(C)** Ili.

*C. spinosa* fruits (6/habitat) were collected from three districts, including Turpan, Karamay, and Ili in Xinjiang Uygur Autonomous Region of China, which were named as groups A, B, and C. The fruits were identified by Dr. Jiang He from the Xinjiang Institute of Materia Medica. The fruits from Turpan were green and medium in size (**Figure 1A**), the fruits from Karamay were the smallest and bottle-green or brown (**Figure 1B**), and the fruits from Ili were the largest and brown (**Figure 1C**). The GPS coordinates and climate information of the habitats can be found in **Table 1**. Turpan had the least precipitation, the most arid climate, and the highest annual average temperature, while Ili had the most humid climate and the lowest annual average temperature, and Karamay had the middle climate index. The contents of N, P, and K in Ili soil were the highest.

**Table 1:** The GPS coordinates and climate information of the habitats.

Factors	Turpan	Karamay	Ili
Sample number	A(A1-A6)	B(B1-B6)	C(C1-C6)
Longitude (degree) Latitude (degree)	89.54 42.83	84.92 45.53	82.15 43.62
Altitude (m)	-61.7031	278.189	823.518
Annual average precipitation (mm)	16.4	108.9	350.2-510
Annual average evaporation (mm)	3000	2692.1	2331
Annual average temperature (°C)	13.9	8.6	5.6
Total nitrogen	0.663	0.08	1.613
Alkeline-N	61	83	126
Olsen-P	31.9	22.89	18.9
Olsen-K	171	199	332
Soil type	Saline anthropogenic alluvial soil	Gypsum ash desert soil	Gypsum gray brown desert soil

### 3.2 Validation of anti-inflammatory function

**Figure 2:**
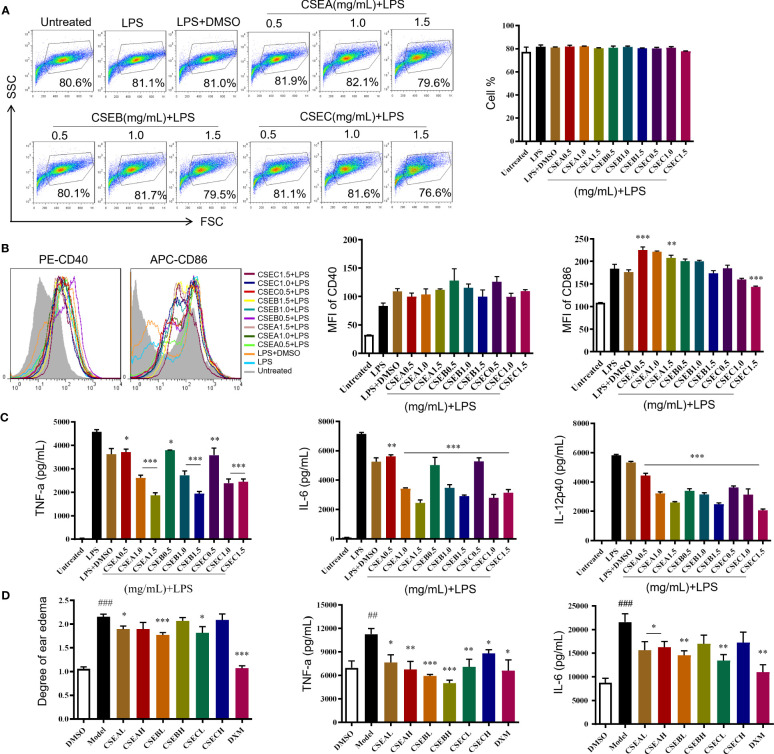
The anti-inflammatory activity of capers from different districts *in vitro* and *in vivo*. **(A–C)** The maturation and the secretion of cytokines of DCs upon CSEs treatment. **(A)** The cell proportion. **(B)** The expression of surface molecular. **(C)** The cytokine production. Data are means ± SE (n = 3). **(D)** Therapeutic effect of CSEs on mouse edema model. Data are means ± SE (n = 5). ##*P* < 0.01, ###*P* < 0.001 compared to DMSO; *P < 0.05, **P < 0.01, ***P < 0.001 compared to LPS/Model.

*C. spinosa* fruits were extracted with 95% ethanol, then the extract was concentrated and lyophilized, named CSE. The yield of the extract was about 24%. CSEs prepared with *C*. *spinosa* fruits from Turpan, Karamay, and Ili were named CSEA, CSEB, and CSEC, respectively. To detect the anti-inflammatory effects of CSE *in vitro*, different concentrations (0.5, 1.0 and, 1.5 mg/mL) of CSEs were used to treat DCs in the presence of LPS. Results of flow cytometry showed that there was no significant change in cell proportion among all groups, suggesting that the selected doses of CSEs did not affect the viability of DCs (**Figure 2A**). Compared with the LPS group, CSEs did not inhibit the expression of surface molecule CD40 induced by LPS, whereas CSEC (1.5 mg/mL) significantly suppressed the expression of CD86 induced by LPS (**Figure 2B**). Three pro-inflammatory cytokines, including TNF-α, IL-6, and IL-12p40, were significantly decreased (**Figure 2C**).

*In vivo*, CSE treatment significantly suppressed the expression of pro-inflammatory cytokines TNF-α and IL-6 and relieved the degree of ear edema in the TPA-induced mouse ear edema model (**Figure 2D**).

These results showed that *C. spinosa* from all three different districts had anti-inflammatory effects, but the effect of fruits from Ili was better than the other two groups in inhibiting DC maturation.

### 3.3 Qualitative and quantitative metabolites

**Figure 3:**
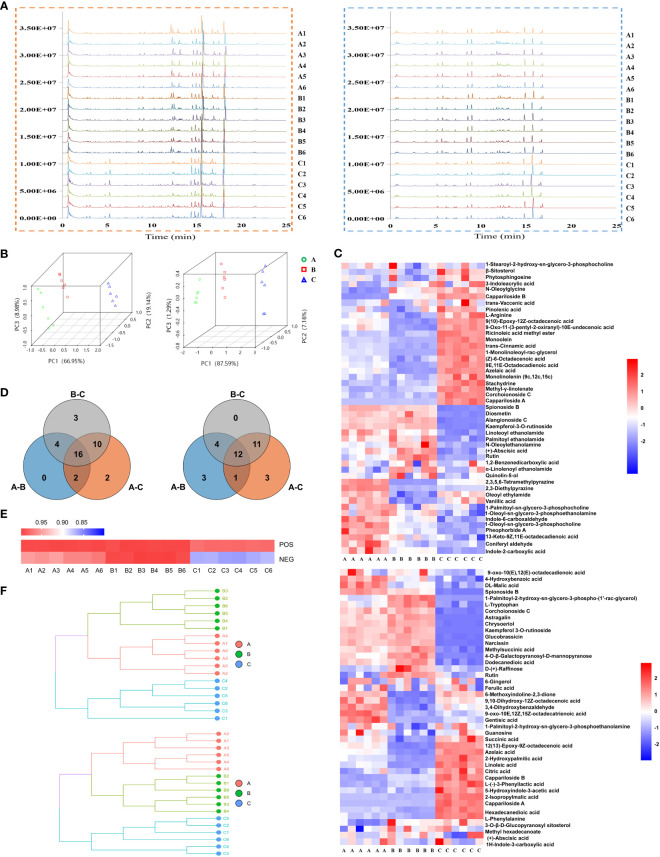
Metabolite’s analysis of *C. spinosa* fruit by ultra-high-performance liquid tandem chromatography quadrupole time of flight mass spectrometry (UHPLC-QTOFMS). **(A)** Chromatographic fingerprint. **(B)** PCA score map. **(C)** HCA of all metabolites. **(D)** Venn map showing the common and differential metabolites. **(E)** Heat map showing the cosine similarity of metabolites. **(F)** Cluster dendrogram showing the cosine similarity of metabolites. In all panels, the positive ion mode was on the left/up and the negative ion mode was on the right/down.

Base peak ion chromatograms of the three groups were shown as fingerprints. There were significant differences among the samples of Ili and the other two districts (**Figure 3A**). A total of 82 compounds were identified, of which 47 were in positive ion mode (**Tables S1**, **S3**) and 43 were in negative ion mode (**Tables S2**, **S4**). Eight compounds were found in both modes. Principal component analysis (PCA) was performed on the 18 samples (6/habitat) (**Figure 3B**) or together with quality control (QC) (**Figure S1**). All samples were within Hotelling’s T-squared ellipse, indicating that the detection system had good stability. In positive ion mode, PC1, PC2, and PC3 were 66.95%, 19.14%, and 8.98%, respectively, while in negative ion mode, PC1, PC2, and PC3 were 87.59%, 7.16% and, 1.29%, respectively (**Figure 3B**). The model of orthogonal partial least squares discriminant analysis (OPLS-DA) was stable and reliable (**Figure S2**). The score plots of PCA and OPLS-DA exhibited an obvious separation among the samples.

The identified compounds were analyzed by hierarchical clustering analysis. The heatmap of hierarchical cluster analysis (HCA) showed that group C was separated from the other two groups (**Figure 3C**). Differential metabolites were screened by the Venn analysis in positive and negative modes, respectively (**Figure 3D**; **Table S5**).

Cosine similarity analysis showed a significant difference among the three groups in negative ion mode, and the similarity value in group C was 86% (**Figure 3E**). The clustering tree revealed that the distance between groups A and B was close, but group C was far from the other two groups (**Figure 3F**).

Stachydrine, ferulic acid, and chrysoeriol were selected to be quantified, and their contents in groups A, B, and C were consistent with the metabolomics (**Figure S3**). These results suggested that the metabolic level of group C was considerably different from that of the other two groups. The metabolomics data were deposited in the OMIX repository, accession number OMIX004470 (https://ngdc.cncb.ac.cn/omix/preview/QoEYJ1oa).

### 3.4 Screening and analyzing of anti-inflammatory compounds

**Figure 4:**
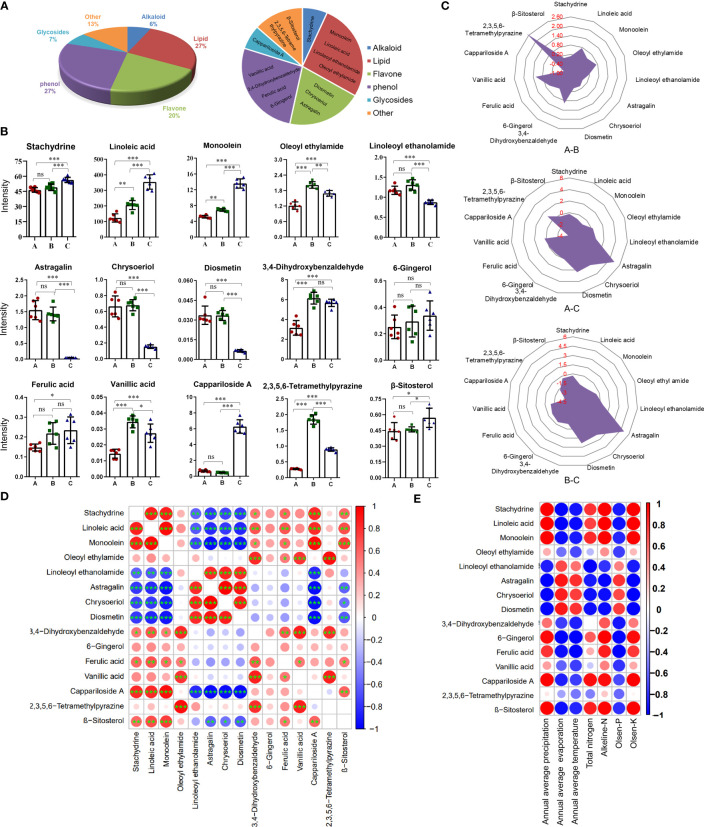
Content differences of 15 anti-inflammatory compounds from the three different districts. **(A)** The pie chart shows the classification of anti-inflammatory compounds. **(B)** Histogram of contents. Data are means ± SE (n = 6). ns: no significance, ^*^P < 0.05, ^**^P < 0.01, ^***^P < 0.001. **(C)** Radar chart showing the log2 (fold change) of anti-inflammatory compounds. **(D)** Bubble chart of correlation analysis for anti-inflammatory compounds. (n = 6). ^*^P < 0.05, ^**^P < 0.01, ^***^P < 0.001. **(E)** Bubble chart of correlation analysis between anti-inflammatory compounds and environmental factors. Data are average.

By retrieving the database, 15 anti-inflammatory compounds were selected. They included one alkaloid, four lipids, three flavones, four phenols, one glycoside, and two other compounds (**Figure 4A**).

Considering the quantitative value (**Figure 4B**) and log2 (fold change) value of the compound (**Figure 4C**), the contents of almost compounds were similar between groups A and C except for oleoyl ethyl amid, 3,4-dihydroxybenzaldehyde, 2,3,5,6-tetramethylpyraz and vanillic acid, but the contents of almost compounds in group A and B were different with that in group C except for 6-gingerol and ferulic acid. The contents of three flavonoids including diosmetin, chrysoeriol, and astragalin in groups A and B were significantly higher than that in group C. The contents of linoleic acid, monoolein, and cappariloside A in group C were significantly higher than that in groups A and B, whereas the content of 2,3,5,6-tetramethylpyrazine in group B was highest. The contents of stachydrine, 6-gingerol, ferulic acid, and β-sitosterol in the three different districts showed no significant difference in the three groups.

The correlation analysis of the quantitative values of metabolites showed that three flavones had a significantly negative correlation with stachydrine, linoleic acid, monoolein, cappariloside A, and β-sitosterol. There was a significantly positive correlation between the three flavones and linoleoyl ethanolamide. Linoleoyl ethanolamide was negatively correlated with stachydrine, linoleic acid, monoolein, and cappariloside A. Stachydrine had a significantly high positive correlation with linoleic acid, monoolein, and cappariloside A. Stachydrine was significantly correlated with 3,4-dihydroxybenzaldehyde, ferulic acid and β-sitosterol. Among the four phenol compounds, ferulic acid, vanillic acid and, 3,4-dihydroxybenzaldehyde had a significant correlation, but the correlation among 6-gingerol and other compounds was weak. The contents of compounds in similar synthetic pathways existed a positive correlation (**Figure 4D**).

The contents of most anti-inflammatory compounds were strongly correlated with environmental factors. High evaporation, high temperature, and high Olsen-P contributed to the accumulation of the three flavones compounds. The trend of linoleoyl ethanolamide was similar to that of flavones. The trends of stachydrine, linoleic acid, monoolein, 6-gingerol, ferulic acid, cappariloside A, and β-sitosterol were opposite to that of flavone. On the other hand, 2,3,5,6-tetramethylpyrazine had a weak correlation with environmental factors (**Figure 4E**).

There was no difference in abscisic acid and tryptophan contents among the three groups, indicating that all the materials were in the same period of fruit development (**Figure S4**). Compounds with consistent response trends to environmental factors also had a strong positive correlation in their content, indicating that the quality variation of *C. spinosa* was caused mainly by environmental factors.

### 3.5 Prediction of anti-inflammatory mechanisms by network pharmacology

**Figure 5:**
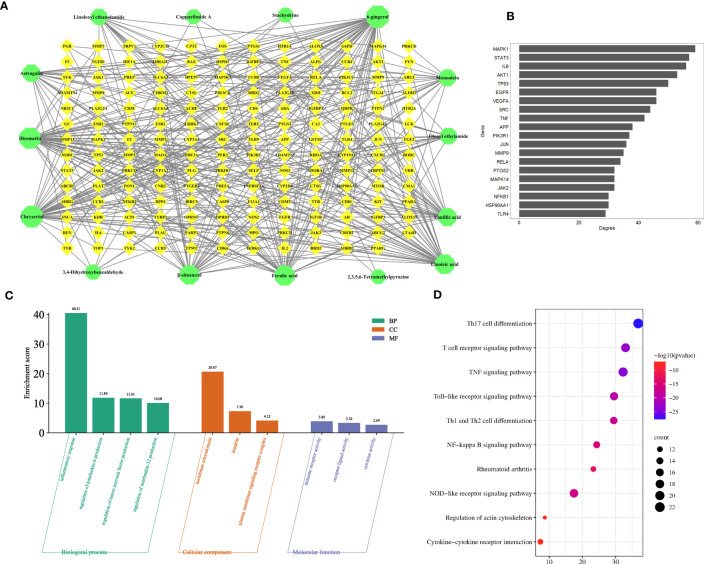
Prediction of the anti-inflammatory mechanism *via* the network pharmacological. **(A)** The compound-target network. **(B)** Core targets. **(C)** GO enrichment analysis. **(D)** KEGG enrichment analysis.

To explore the anti-inflammatory mechanism of *C. spinosa* fruit, the above 15 anti-inflammatory compounds were used for network pharmacological analysis. 564 targets of compounds were collected, and 1,196 human targets of rheumatoid arthritis (RA) were obtained. Thus, after intersection analysis, 167 common targets were acquired (**Figure S5**). The common targets were imputed into STRING to build the protein-protein interaction (PPI) network. Subsequently, the interaction network was constructed for illustrating the interactive relationship between compounds and targets (**Figure 5A**). Based on degree value, the top 20 were the core targets including MAPK1, STAT3, IL-6, TNF, VEGFA (vascular endothelial growth factor A), SRC, MMP9, and JUN (**Figure 5B**).

In addition, the common targets were subjected to GO and KEGG enrichment analysis, and the terms associated with RA of GO mainly focused on the regulation of interleukin-6 production, regulation of tumor necrosis factor production, regulation of interleukin-12 production, dendrite, immune receptor activity, cytokine activity, etc. (**Figure 5C**). KEGG pathways mainly focused on the TNF signaling pathway, NF-kappa B signaling pathway, Cytokine-cytokine receptor interaction, etc. (**Figure 5D**).

These results suggested that *C. spinosa* plays an anti-inflammatory role by affecting cytokine expression and lymphocyte differentiation.

### 3.6 Screening of anti-inflammatory compounds *in vitro*

**Figure 6:**
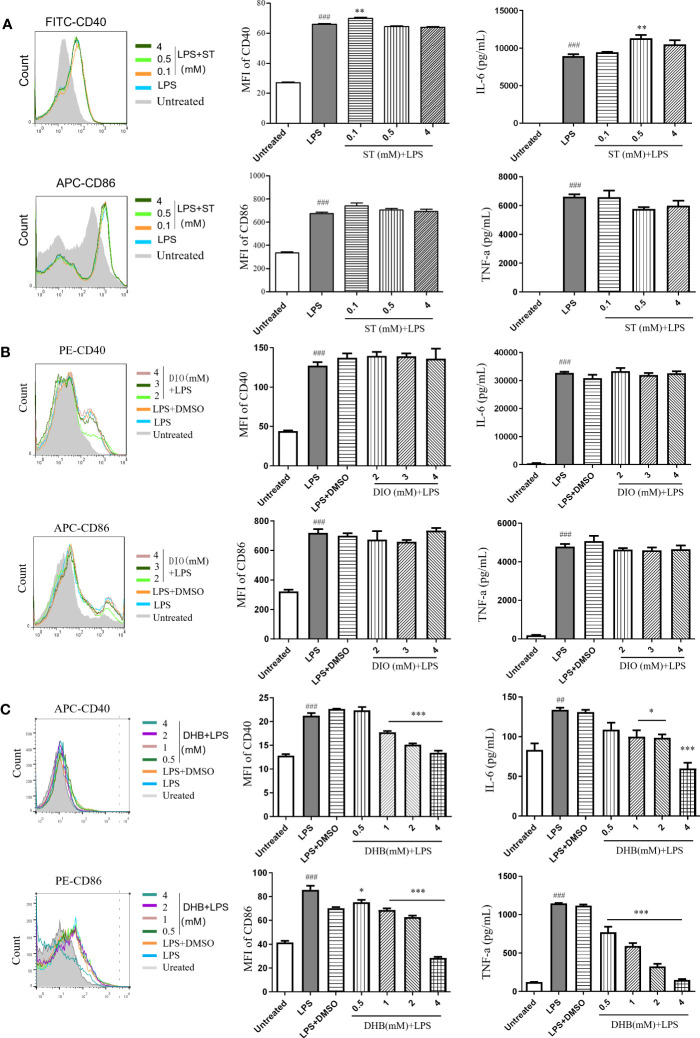
Anti-inflammatory effects of three compounds *in vitro*. **(A)** Stachydrine (ST). **(B)** Diosmetin (DIO). **(C)** 3,4-Dihydroxybenzaldehyde (DHB). Data are means ± SE (n = 3). *^##^P* < 0.01, *^###^P* < 0.001 compared to Untreated; ^*^ *P* < 0.05, ^**^ *P* < 0.01, ^***^ *P* < 0.001 compared to LPS.

To identify key active compounds in CSE that inhibit the maturation of LPS-induced DC, we examined the effect of stachydrine (ST), diosmetin (DIO), and 3,4-dihydroxybenzaldehyde (DHB) to treat DCs in the presence of LPS. Sachydrine and diosmetin did not inhibit the expression of costimulatory molecules (CD40 and CD86) and the secretion of pro-inflammatory cytokines IL-6 and TNF-α (**Figures 6A, B**). 3,4-dihydroxybenzaldehyde significantly suppressed the expression of CD40 and IL-6 induced by LPS at the concentration of 1 mM, and it significantly suppressed the expression of CD86 and TNF-α at the concentration of 0.5 mM (**Figure 6C**). The results showed that phenols were the main compounds that inhibited DCs maturation and secretion of pro-inflammatory cytokines in capers.

### 3.7 Identifying key genes in phenols biosynthetic pathway

Transcriptome data of *C. spinosa* fruits were analyzed. PCA showed PC1, PC2, and PC3 were 49.31%, 17.50%, and 9.04%, respectively, and all samples were within the Hotelling’s T-squared ellipse. Group C was significantly separated from the other two groups (**Figure S6A**). Volcano Analysis showed that 2,432 genes were up-regulated, and 1,705 genes were down-regulated in group A and group B, 9,104 genes were up-regulated, and 4,209 genes were down-regulated in group A and group C, 4,872 genes were up-regulated, and 2.320 genes were down-regulated in group B and group C (**Figure S6B**). HCA and Venn’s analysis showed that group C was significantly different from the other two groups (**Figures** **S6C, D**). The enrichment differential pathways were concentrated in biosynthesis and metabolism pathways between groups A and C (**Figure S7**). These data indicated that group C was significantly different from the other two groups at the transcriptional level. These transcript data were deposited into the NCBI database with identifier number PRJNA778809 (https://www.ncbi.nlm.nih.gov/sra/PRJNA778809).

**Figure 7:**
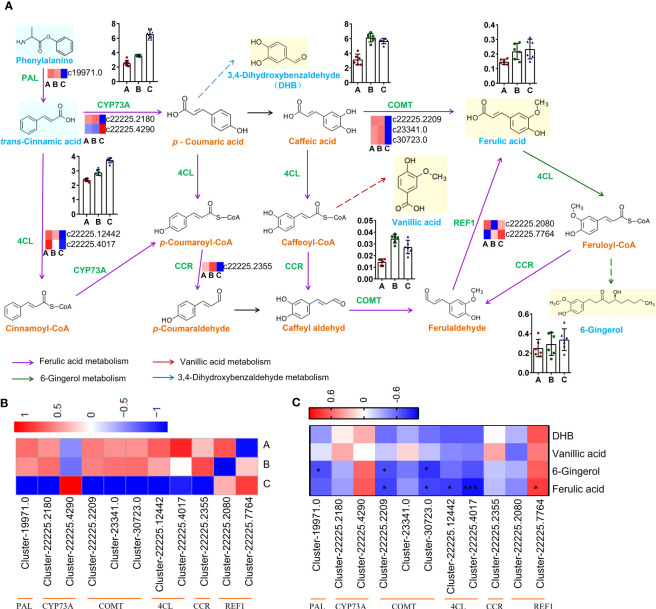
Pathways and genes involved in the biosynthesis of phenols. **(A)** Changes in gene expression and compound accumulation in the synthetic pathway. **(B)** Heatmap showing the expression profiles of genes related to phenols from three districts. **(C)** Heat map showing correlation analysis of compound content and gene expression level. *P < 0.05, ***P < 0.001.

The biosynthetic pathway was constructed based on three KEGG pathways: ‘Phenylpropanoid biosynthesis (Ko00940)’, ‘Stilbenoid, diarylheptanoid and gingerol biosynthesis (Ko00945)’, ‘Biosynthesis of alkaloids derived from shikimate pathway (Ko01063)’ (**Figure 7A**).

The genes encoding PAL (Cluster-19971.0), CYP73A (Cluster-22225.2180), COMT (Cluster-22225.2209, Cluster-23341.0, Cluster-30723.0), 4CL (Cluster-22225.12442, Cluster- 22225.4017), CCR (Cluster-22225.2355) were down-regulated in group C, but the genes encoding CYP73A (Cluster-22225.4290) and REF1 (Cluster-22225.2080, Cluster-22225.7764) were up-regulated in group C (**Figure 7B**). The qRT−PCR results showed good consistency with the RNA-Seq data. The qRT−PCR results indicated that the expression of CYP73A (Cluster-22225.2180) in groups A and B was significantly higher than that in group C, while the expression of CYP73A (Cluster-22225.4290) in group C was significantly higher than that in the other two groups. REF1 (Cluster-22225.7764) was highly expressed in all three groups (**Figure S8**).

The expression level of REF1 (Cluster-22225.7764) indicated a strong positive correlation with ferulic acid content (r = 0.7667, *P* = 0.0214), which might be the key gene promoting ferulic acid biosynthesis in *C. spinosa* (**Figure 7C**). These results indicated that *C. spinosa* fruits from different districts affected the biosynthesis of secondary metabolites by regulating gene transcription.

## 4 Material and methods

### 4.1 Materials and regents

The fruits of *C. spinosa* were collected from June to July 2019 from Turpan, Karamay, and Ili in Xinjiang Uygur Autonomous Region of China. The fruits were immediately frozen in liquid nitrogen and then stored at -80°C. The identification was carried out by Dr. Jiang He of Xinjiang Institute of Materia Medica.

BALB/c mice (20 ± 5 g) aged six weeks old were obtained from the experimental animal center of the Xinjiang Medical University (Urumqi, China). Mice were allowed free access to distilled water and standard food, mice room under conditions of maintained temperature (24~26°C) and humidity (69~71%) as well as a 12:12 h light-dark cycle. All experimental animal protocols were reviewed and approved by the Ethics Committee of Xinjiang University for the use of Laboratory Animals.

### 4.2 Preparation of *C. spinosa* fruits ethanol extracts

First, the dried fruits of *C. spinosa* were ground into a powder and passed through a 40-mesh sieve. Then, 50 g of the powder was mixed with 95% ethanol in a ratio of 1:6 (w/v). The mixture was ultra-sonicated for 20 minutes, followed by extraction for 2 hours in a water bath at 60°C. This extraction process was repeated three times, and the extracts were mixed. The extract was concentrated using rotary evaporation. Then, the extract was subjected to lyophilization and named ‘CSE’.

### 4.3 Detection of anti-inflammatory effect *in vitro*

Immature DCs were induced from bone marrow cells of BALB/c mice by Granulocyte-macrophage colony-stimulating factor (GM-CSF, Peprotech, USA) (Aipire et al., 2017). The cells were cultured in RPMI-1640 medium containing 10% heat-inactivated fetal bovine serum (FBS), 100 units/ml penicillin-streptomycin, 50μM β-mercaptoethanol and 20 ng/ml GM-CSF. Subsequently, they were placed in a 5% CO_2_, 37°C incubator. The cells were collected on the seventh day, cultured at a concentration of 1 × 10^6^/mL on a 24-well plate, and treated with different concentrations of CSEs or standards in the presence of 80 ng/mL LPS for 12h. The cells were collected and stained using the mAbs, including anti-mouse CD40, and anti-mouse APC-CD86. All samples were detected on FACSCalibur (BD Biosciences, USA), and the data were analyzed by the FlowJo software (v7.6). The cell supernatant was collected, and the cytokines (TNF-α, IL-6, and IL-12p40) were measured by ELISA kit. *OD*_450_ was measured using a microplate reader (Bio-Rad, USA).

### 4.4 Treatment of TPA-induced mouse ear edema model

TPA was used to induce the ear edema model in mice. The 45 mice were randomly divided into a control group (DMSO), model group (TPA, 75 μg/kg), positive control group (dexamethasone, DXM, 2.5 mg/kg), low-dose group (200 mg/kg) and high-dose group (500 mg/kg), with five mice in each group. The medicine was applied to the right ear for 6 h, ear edema degree of mice was calculated by right ear mass (mg)/left ear mass (mg), and the cytokines (TNF-α and IL-6) were measured by ELISA kit.

### 4.5 Metabolites extraction and LC-MS/MS analysis

A 100 mg sample was extracted with 500 μL of 80% methanol. 2-Chloro-L-phenylalanine (1000:10) was used as the internal standard. After 30 s vortex, the samples were homogenized at 45 Hz for 4 min. Then the extracts were ultra-sonicated for 1 hour in the ice bath. The extracts were left at -20°C for 1 h and centrifuged at 12000 rpm for 15 min at 4°C. The supernatant was aspirated and filtered through a 0.22 μm filter membrane and set aside.

LC-MS/MS analysis was detected on an AB SCIEX Triple TOF 5600 mass spectrometer coupled with a Shimadzu Nexera UPLC LC-30A system with a Waters UPLC BEH C18 (1.7 μm × 2.1 × 100 mm) column. The flow rate was 0.4 mL/min, and the sample injection volume was 3 μL. The mobile phases were 0.1% formic acid aqueous solution (A) and 0.1% formic acid acetonitrile solution (B). The elution gradients were: 0-3.5 min, 95-85% A; 3.5-6 min, 85-70% A; 6-6.5, 70-70% A; 6.5-12 min, 70-30% A; 12-12.5 min, 30-30% A; 12.5-18 min, 30-0% A; 18-22 min, 0% A. Data were collected using Analyst TF 1.7 software to view and process.

### 4.6 Differential metabolite analysis

The raw data were imported into Progenesis QI software for retention time correction, peak identification, peak extraction, peak integration, and peak alignment, and the corresponding TCM metabolic database was established. Substance identification of the unknown components was performed using the self-built mass spectrometry database. Multivariate statistical analysis, including PCA, and OPLS-DA, was performed using SIMCA software (V15.0.2, Sartorius Stedim Data Analytics AB, Umea, Sweden). Also, R software or GraphPad Prism software (V8.0, La Jolla, CA, USA) was used HCA, Venn analysis, cosine similarity analysis, radar plot, clustering tree, pie chart, histogram, and Pearson correlation analysis were performed in combination with univariate analysis for metabolite accumulation patterns between different samples.

### 4.7 Validation of metabolomic data by UHPLC-QTOF-MS

Metabolites were extracted according to 2.2. Stachydrine, ferulic acid, and chrysinol (HPLC ≥ 98%, Yuanye, China) were dissolved in with methanolic solution, respectively. The prepared extracts were each taken 400 μL, mixed to prepare mixed standard solutions and a series of mixed standard solutions of different concentrations were prepared by stepwise dilution with methanol. The mass spectrometer apparatus and elution conditions were the same as in 3.4.

### 4.8 Analysis of network pharmacology

The compounds with anti-inflammatory functions were screened from metabolites for network pharmacological analysis. The chemical targets were screened by TCMSP databases (http://lsp.nwu.edu.cn/tcmsp.php), Swiss Target Prediction Database (http://www.swisstargetprediction.ch/), SEA databases (https://sea.bkslab.org/). The disease targets were screened by GeneCards (https://www.genecards.org), and OMIM databases (https://www.omim.org/). Furthermore, the keyword ‘rheumatoid arthritis’ was used to retrieve all targets under the condition of ‘Homo sapiens’. Venny software (v2.1.0) was used to intersect anti-inflammatory component targets and disease targets, which were named ‘the common targets’. Then, the common targets of RA and active ingredients were used to construct the PPI network by the STRING database (https://string-db.org/cgi/input.pl). The results were visualized using Cytoscape software (v3.6.1). The top 20 targets with degree value in the PPI network were named ‘core targets’. The R Project was used to visualize core targets. GO enrichment analysis of common targets was performed by Metascape (http://metascape.org).

### 4.9 Extraction RNA and analysis of transcriptomics

Total RNA was extracted using TRIzol reagent, and genomic DNA was removed using DNase I (Takara) according to the manufacturer’s instructions. RNA quality and quantification were measured using a 2100 Bioanalyser (Agilent) and an ND-2000 (Nano-Drop Technologies) RNA-seq transcriptome libraries were then prepared from total RNA (1 μg) using the TruSeqTM RNA Sample Preparation Kit (Illumina, San Diego, CA, USA). The expression levels of each transcript were calculated according to the mapping reads per million exon kilobase fragment method to identify these DEGs in different samples. EdgeR (Empirical Analysis of Digital Gene Expression in R) was used for differential expression analysis and NR (NCBI non-redundant protein sequences) was used to annotate the assembled unigenes. In addition, Gene Ontology (GO) and Kyoto Encyclopedia of Genes and Genomes (KEGG) functional enrichment analyses were performed for these DEGs using Goatools (https://github.com/tanghaibao/Goatools) and KOBAS (http://kobas.cbi.pku.edu.cn/home.do), respectively.

### 4.10 Correlation analysis mining anti-inflammatory compounds and genes

Based on the KEGG pathway analysis (https://www.kegg.jp/kegg/pathway.html), genes related to the synthesis of anti-inflammatory compounds were screened. The key genes were identified by Spearman correlation analysis using the GraphPad Prism 8 software.

### 4.11 Validation of RNA−seq data by qRT−PCR

Total RNA was reverse transcribed using the reverse transcription kit. Then, qPCR amplification was performed on the qTOWER3G qPCR instrument. The primer sequences are shown in **Table S1**.

### 4.12 Statistical analysis

Statistical significance analysis was performed using one-way ANOVA or t-test and computed using GraphPad Prism software. A value of *P* < 0.05 was statistically significant.

## 5 Discussion

In this study, we compared the fruit quality differences of capers from different districts in Xinjiang through metabolome and transcriptome analysis and detected their anti-inflammatory activity *in vitro* and *in vivo*. The fruits from Ili were significantly different from Turpan and Karamay in metabolism and transcription levels. In addition, fruits from all three different districts inhibited the expression of pro-inflammatory cytokines, but ethanol extract of caper from Ili significantly inhibited the expression of CD86 on DCs induced by LPS. There were significant differences in the different districts, morphology, metabolites, gene expression level, and anti-inflammatory activity of capers from the three districts.

*C. spinosa* fruit extract rich in flavonoids, indoles, and phenolic acids, effectively inhibited carrageenan-induced paw edema in mice (Zhou et al., 2010). *C. spinosa* fruit ethanol extracts significantly inhibited the secretions of IL-12p40, IL-6, IL-1β, and TNF-α in LPS-induced DC (Azeguli et al., 2017). *C. spinosa* leaf alcoholic extract significantly decreased immune cell infiltration, vasodilatation, and dermis thickness in the inflammatory site by inhibiting cytokine gene expression, including IFN-γ, IL-17, and IL-4 in the contact hypersensitivity mice. The extract contained saponins, flavonoids, and alkaloids (Azhary et al., 2017). 70% alcohol extract of *C. spinosa* aerial parts significantly reduced the levels of TNF-α, COX-2, IL-1β, and IL-6 in the LPS-induced inflammation in microglia (Rahimi et al., 2020). Pro-inflammatory cytokines IL-6, TNF-α, and IL-1 played an important pathogenic role in inflammatory processes such as RA (Kaneshiro et al., 2019). The results of this study also confirmed that *C. spinosa* extract could treat inflammation by inhibiting the expression of pro-inflammatory cytokines both *in vitro* and *in vivo*. Thus, fruits from all three districts can be used in the treatment of inflammatory diseases.

At present, most studies on the anti-inflammatory effects and mechanisms of *C. spinosa* fruit are based on experimental animal models. However, it remains uncertain whether comparable positive effects can be achieved in the treatment of human diseases. We will further verify the anti-inflammatory effect of *C. spinosa* fruit on human cells. These results will lay the foundation for clinical trials of the fruit for the treatment of inflammatory diseases.

However, different geographical locations greatly influence the accumulation of active compounds and their function in *C. spinosa.* In this study, we found that the content of secondary metabolites of *C. spinosa* fruits from three different districts was significantly different. Consistently, the analysis of different samples from various regions of Sardinia (Italy) revealed qualitative and quantitative differences in the content of flavonoids, glucosinolates, anthocyanins, and phenolic acids (Maldini et al., 2016). The *C. spinosa* bud samples of Morocco, Turkey, and Italy were analyzed, and the results showed that the content of phenol in the Morocco sample was the highest, and that of rutin in the Italian decoction was the highest. Significantly, the highest acetylcholinesterase inhibitory activity was observed in Turkish and Moroccan samples, and the best butyrylcholinesterase inhibitory effects were detected in the Italian samples (Stefanucci et al., 2018). Therefore, the chemical composition of *C. spinosa* with different geographical distributions changed significantly, which endowed them with various biological activities. Among the anti-inflammatory compounds, the flavonoids in fruits from Turpan were the highest, while those in fruits from Ili were the lowest. The biosynthesis and accumulation of flavonoids were related to drought stress (Nakabayashi et al., 2014; Yang et al., 2020). The humid climate in the Ili region was not conducive to the accumulation of flavonoids.

The extracts from all three districts reduced TNF-α and IL-6 both *in vitro* and *in vivo*. However, only a few authors reported the inhibition of TNF-α and IL-6 by flavonoids (Peluso et al., 2015). DCs are professional antigen-presenting cells, link innate and adaptive immune responses. They regulate T cell homeostasis and inflammatory response by expressing costimulatory molecules and releasing cytokines (Waisman et al., 2017). However, ethanol extract of caper from Ili had the best inhibitory effect on the expression of DC surface molecule CD86. Therefore, flavonoids may not be the main component of *C. spinosa* fruits in inhibiting DC maturation. Our experimental results showed that diosmetin had no anti-inflammatory activity at the concentration of 4 mM, which supported the above conclusions.

The contents of stachydrine were higher in 15 anti-inflammatory compounds, and there was little difference in the contents among the three districts. But stachydrine also had no anti-inflammatory activity. Therefore, the anti-inflammatory components in capers need to be further verified by subsequent experiments. Ferulic acid reduced TNF-α, IL-1β, IL-6, IL-23, and MMP9, and increased TGF-β in inflammatory conditions (Doss et al., 2018; Ganesan and Rasool, 2019; Yin et al., 2019; Zhu et al., 2020). On the other hand, 6-gingerol inhibited COX-2, IL-17, IL-6, IL-1β, IL-8, and TNF-α, and increased IL-10 to treat inflammation (Kim et al., 2005; Saha et al., 2016; Zhang and Zheng, 2018; Zhang et al., 2018; Liu et al., 2020; Sheng et al., 2020). 3,4-Dihydroxybenzaldehyde was a phenolic compound with high content in capers. Our results showed that it significantly inhibited DCs maturation and secretion of pro-inflammatory cytokines. These studies suggested that phenols had good anti-inflammatory activity.

Plants respond to environmental changes by regulating gene expression and thus affect metabolic processes. *Cistanche deserticola* was investigated and the 12 key genes involved in PhGs biosynthesis were found to be differentially expressed among three ecotypes (Sun et al., 2020). The content of starting compound phenylalanine was the highest in group C. However, due to the down-regulated expression of most genes in the synthetic pathway, there was no difference in the content of the final product 6-gingerol among the three groups. As an intermediate product, ferulic acid did not accumulate in the three groups. According to the content of compounds, the biosynthetic pathway of 3,4-dihydroxybenzaldehyde played a dominant role.

## 6 Conclusion

In this study, we found there were significant differences in the morphology, metabolites, gene expression level, and anti-inflammatory activity of capers from the three different districts. All the 95% ethanol extracts from the three districts showed remarkable anti-inflammatory effects, but the anti-inflammatory effects of *Capparis spinosa* L. vary by origin. The extract from Ili significantly inhibited the expression levels of CD86 on DCs induced by LPS. Phenols were the main compounds that inhibited DCs maturation and secretion of pro-inflammatory cytokines in capers. The gene of REF1 (Cluster-22225.7764) might be the key gene promoting ferulic acid biosynthesis in *C. spinosa*.

## Data availability statement

The metabolomics data presented in the study are deposited in the OMIX repository, accession number OMIX004470. The transcript data presented in the study are deposited in the NCBI repository, accession number PRJNA778809.

## Ethics statement

The animal study was reviewed and approved by Ethics Committee of Xinjiang University, Xinjiang University (XJUAE-2019-017).

## Author contributions

XL, AA, XW, and JL designed the research and analyzed the data. XL, AA, YD, PH, and LM performed the experiments. JL, XW, and XL wrote the manuscript. All authors contributed to the article and approved the submitted version.
