# Supplementary material for: Quality variation and biosynthesis of anti-inflammatory compounds for Capparis spinosa based on the metabolome and transcriptome analysis
Source: Front Plant Sci. 2023 Jul 17;14:1224073. doi: 10.3389/fpls.2023.1224073 (PMC10388242; doi:10.3389/fpls.2023.1224073)
Supplement: Supplementary file 2 [file DataSheet_2.docx]

Table S3. The components in positive ion modes

| **NO.** | **Identification** | **m/z** | **Rt (min)** | **Adducts** | **Formula** | **Molecular weight (Da)** |
| --- | --- | --- | --- | --- | --- | --- |
| 1 | Stachydrine | 144.1013 | 0.66 | M+H | C_7_H_13_NO_2_ | 144.19 |
| 2 | (Z)-6-Octadecenoic acid | 265.2519 | 16.8 | M+H-H2O, M+Na, M+H | C_18_H_34_O_2_ | 282.46 |
| 3 | L-Arginine | 175.1186 | 0.57 | M+H | C_6_H_14_N_4_O_2_ | 174.20 |
| 4 | Pinolenic acid | 279.2313 | 14.86 | M+H-H2O, M+H | C_18_H_30_O_2_ | 278.43 |
| 5 | trans-Cinnamic acid | 166.0853 | 1.54 | M+H-H2O, M+NH4, M+H | C_9_H_8_O_2_ | 148.16 |
| 6 | 3-Indoleacrylic acid | 188.0704 | 2.32 | M+H, M+NH4 | C_11_H_9_NO_2_ | 187.19 |
| 7 | Monolinolenin (9c,12c,15c) | 353.2678 | 14 | M+H, M+Na, M+NH4, M+H-H2O | C_21_H_36_O_4_ | 352.51 |
| 8 | β-Sitosterol | 397.3814 | 18.84 | M+H-H2O | C_29_H_50_O | 414.71 |
| 9 | 9E,11E-Octadecadienoic acid | 263.2363 | 15.76 | M+H-H2O, M+H, M+Na | C_18_H_32_O_2_ | 280.45 |
| 10 | 13-Keto-9Z,11E-octadecadienoic acid | 295.2262 | 12.87 | M+H-H2O, M+H | C_18_H_30_O_3_ | 294.43 |
| 11 | Phytosphingosine | 318.2995 | 11.5 | M+H-H2O, M+H | C_18_H_39_NO_3_ | 317.51 |
| 12 | α-Linolenoyl ethanolamide | 322.2734 | 13.01 | M+H, M+Na | C_20_H_35_NO_2_ | 321.50 |
| 13 | Quinolin-5-ol | 146.0596 | 2.32 | M+H | C_9_H_7_NO | 145.16 |
| 14 | N-Oleoylglycine | 340.284 | 15.15 | M+H, M+Na | C_20_H_39_NO_2_ | 325.53 |
| 15 | 9(10)-Epoxy-12Z-octadecenoic acid | 279.2313 | 12.45 | M+H-H2O | C_18_H_32_O_3_ | 296.44 |
| 16 | Oleoyl ethylamide | 310.3096 | 16.46 | M+H, M+Na | C_20_H_39_NO | 309.53 |
| 17 | Corchoionoside C | 387.2026 | 3.75 | M+H | C_19_H_30_O_8_ | 386.44 |
| 18 | 1-Palmitoyl-sn-glycero-3-phosphocholine | 496.3383 | 12.87 | M+H, M+Na | C_24_H_50_NO_7_P | 495.63 |
| 19 | 1-Stearoyl-2-hydroxy-sn-glycero-3-phosphocholine | 524.3693 | 14.38 | M+H, M+Na | C_26_H_54_NO_7_P | 523.68 |
| 20 | Linoleoyl ethanolamide | 324.289 | 13.93 | M+H, M+Na | C_20_H_37_NO_2_ | 323.51 |
| 21 | Ricinoleic acid methyl ester | 335.2557 | 15.13 | M+H-H2O, M+Na | C_19_H_36_O_3_ | 312.49 |
| 22 | (+)-Abscisic acid | 247.1324 | 7.12 | M+H-H2O, M+Na | C_15_H_20_O_4_ | 264.32 |
| 23 | 1-Oleoyl-sn-glycero-3-phosphoethanolamine | 480.3069 | 13.14 | M+H, M+Na | C_23_H_46_NO_7_P | 478.58 |
| 24 | Monoolein | 357.2991 | 16.07 | M+H, M+NH4, M+Na, M+H-H2O | C_21_H_40_O_4_ | 56.54 |
| 25 | Rutin | 611.1597 | 5.16 | M+H, M+Na | C_27_H_30_O_16_ | 610.52 |
| 26 | Methyl-γ-linolenate | 293.2468 | 16.78 | M+H | C_19_H_32_O_2_ | 292.46 |
| 27 | 9-Oxo-11-(3-pentyl-2-oxiranyl)-10E-undecenoic acid | 311.2208 | 11.61 | M+H | C_18_H_30_O_4_ | 310.43 |
| 28 | 1-Monolinoleoyl-rac-glycerol | 377.2652 | 14.97 | M+H, M+Na, M+NH4, M+H-H2O | C_21_H_38_O_4_ | 354.52 |
| 29 | Azelaic acid | 211.0939 | 6.24 | M+H-H2O, M+Na | C_9_H_16_O_4_ | 188.22 |
| 30 | Palmitoyl ethanolamide | 300.2891 | 14.68 | M+H, M+Na, M+H-H2O | C_18_H_37_NO_2_ | 299.49 |
| 31 | Cappariloside B | 519.1579 | 2.28 | M+NH4, M+Na | C_22_H_28_N_2_O_11_ | 496.46 |
| 32 | 1,2-Benzenedicarboxylic acid | 149.0228 | 13.14 | M+H-H2O | C_8_H_6_O_4_ | 166.13 |
| 33 | Vanillic acid | 169.0492 | 3.26 | M+H | C_8_H_8_O_4_ | 168.15 |
| 34 | Coniferyl aldehyde | 179.0699 | 5.85 | M+H | C_10_H_10_O_3_ | 178.19 |
| 35 | Indole-6-carboxaldehyde | 146.0595 | 5.6 | M+H | C_9_H_7_NO | 145.16 |
| 36 | N-Oleoylethanolamine | 326.3046 | 15.04 | M+H, M+Na | C_20_H_39_NO_2_ | 325.53 |
| 37 | 1-Oleoyl-sn-glycero-3-phosphocholine | 522.3537 | 13.19 | M+H, M+Na | C_26_H_52_NO_7_P | 521.67 |
| 38 | Alangionoside C | 411.1975 | 4.47 | M+Na | C_19_H_32_O_8_ | 388.45 |
| 39 | Spionoside B | 425.1774 | 2.69 | M+Na | C_19_H_30_O_9_ | 402.45 |
| 40 | trans-Vaccenic acid | 283.2625 | 13.52 | M+H-H2O, M+H | C_18_H_34_O_2_ | 282.46 |
| 41 | Cappariloside A | 357.1056 | 2.8 | M+NH4, M+Na, M+H | C_16_H_18_N_2_O_6_ | 334.32 |
| 42 | Pheophorbide A | 593.2734 | 16.12 | M+H, M+Na | C_35_H_36_N_4_O_5_ | 592.68 |
| 43 | Diosmetin | 301.0706 | 8.22 | M+H | C_16_H_12_O_6_ | 300.26 |
| 44 | Indole-2-carboxylic acid | 162.0544 | 3.05 | M+H | C_9_H_7_NO_2_ | 161.16 |
| 45 | Kaempferol 3-O-rutinoside | 595.1648 | 5.69 | M+H, M+Na | C_27_H_30_O_15_ | 594.52 |
| 46 | 2,3,5,6-Tetramethylpyrazine | 137.1067 | 2.83 | M+H | C_8_H_12_N_2_ | 136.19 |
| 47 | 2,3-Diethylpyrazine | 137.1068 | 4.96 | M+H | C_8_H_12_N_2_ | 136.19 |

Table S4. The components in negative ion modes

| **NO.** | **Identification** | **m/z** | **Rt(min)** | **Adducts** | **Formula** | **Molecular weight (Da)** |
| --- | --- | --- | --- | --- | --- | --- |
| 1 | Linoleic acid | 279.2324 | 15.77 | M-H, M+FA-H | C_18_H_32_O_2_ | 280.45 |
| 2 | 12(13)-Epoxy-9Z-octadecenoic acid | 295.2274 | 12.45 | M-H | C_18_H_32_O_3_ | 296.44 |
| 3 | L-Phenylalanine | 164.0717 | 1.53 | M-H | C_9_H_11_NO_2_ | 165.19 |
| 4 | Succinic acid | 117.0192 | 0.93 | M-H | C_4_H_6_O_4_ | 118.09 |
| 5 | Hexadecanedioic acid | 285.2066 | 11.69 | M-H | C_16_H_30_O_4_ | 286.41 |
| 6 | Azelaic acid | 187.0975 | 6.26 | M-H | C_9_H_16_O_4_ | 188.22 |
| 7 | Citric acid | 191.0198 | 0.84 | M-H | C_6_H_8_O_7_ | 192.12 |
| 8 | 9,10-Dihydroxy-12Z-octadecenoic acid | 313.2379 | 11.26 | M-H | C_18_H_34_O_4_ | 314.46 |
| 9 | Cappariloside B | 495.1605 | 2.28 | M-H, M+FA-H | C_22_H_28_N_2_O_11_ | 496.46 |
| 10 | 6-Methoxyindoline-2,3-dione | 222.0409 | 1.39 | M+FA-H | C_9_H_7_NO_3_ | 177.16 |
| 11 | 5-Hydroxyindole-3-acetic acid | 190.051 | 4.16 | M-H | C_10_H_9_NO_3_ | 191.18 |
| 12 | L-(-)-3-Phenyllactic acid | 165.0557 | 4.78 | M-H | C_9_H_10_O_3_ | 166.17 |
| 13 | 1-Palmitoyl-2-hydroxy-sn-glycero-3-phosphoethanolamine | 452.2765 | 12.83 | M-H | C_21_H_44_NO_7_P | 453.55 |
| 14 | 3,4-Dihydroxybenzaldehyde | 137.0242 | 2.56 | M-H | C_7_H_6_O_3_ | 138.12 |
| 15 | 2-Hydroxypalmitic acid | 271.2274 | 14.98 | M-H | C_16_H_32_O_3_ | 272.42 |
| 16 | Guanosine | 282.0844 | 0.89 | M-H | C_10_H_13_N_5_O_5_ | 283.24 |
| 17 | DL-Malic acid | 133.0141 | 0.66 | M-H2O-H, M-H | C_4_H_6_O_5_ | 134.09 |
| 18 | Ferulic acid | 193.0504 | 5.03 | M-H | C_10_H_10_O_4_ | 194.18 |
| 19 | 2-Isopropylmalic acid | 175.0613 | 2.9 | M-H | C_7_H_12_O_5_ | 176.17 |
| 20 | 9-oxo-10(E),12(E)-octadecadienoic acid | 293.2116 | 13.05 | M-H | C_18_H_30_O_3_ | 294.43 |
| 21 | 3-O-β-D-Glucopyranosyl sitosterol | 621.4355 | 18.85 | M+FA-H | C_35_H_60_O_6_ | 576.85 |
| 22 | L-Tryptophan | 203.0827 | 2.33 | M-H | C_11_H_12_N_2_O_2_ | 204.23 |
| 23 | 9-oxo-10E,12Z,15Z-octadecatrienoic acid | 291.1961 | 12.25 | M-H | C_18_H_28_O_3_ | 292.41 |
| 24 | Methylsuccinic acid | 131.0349 | 1.69 | M+FA-H | C_17_H_34_O_2_ | 270.45 |
| 25 | 6-Gingerol | 293.1756 | 10.33 | M-H | C_17_H_26_O_4_ | 294.39 |
| 26 | (+)-Abscisic acid | 263.1287 | 7.14 | M-H | C_15_H_20_O_4_ | 264.32 |
| 27 | Rutin | 609.1454 | 5.17 | M-H | C_27_H_30_O_16_ | 610.52 |
| 28 | 1H-Indole-3-carboxylic acid | 160.0407 | 5.63 | M-H | C_9_H_7_NO_2_ | 161.16 |
| 29 | Methyl hexadecanoate | 315.2533 | 11.91 | M+FA-H | C_17_H_34_O_2_ | 270.45 |
| 30 | 4-Hydroxybenzoic acid | 137.0244 | 6.08 | M-H | C_7_H_6_O_3_ | 138.12 |
| 31 | Dodecanedioic acid | 229.1446 | 9.13 | M-H | C_12_H_22_O_4_ | 230.30 |
| 32 | Spionoside B | 447.1861 | 2.7 | M-H, M+FA-H | C_19_H_30_O_9_ | 402.45 |
| 33 | Gentisic acid | 153.0193 | 1.85 | M-H | C_7_H_6_O_4_ | 154.12 |
| 34 | Corchoionoside C | 431.1909 | 3.77 | M+FA-H | C_19_H_30_O_8_ | 386.44 |
| 35 | Cappariloside A | 333.1087 | 2.79 | M-H, M+FA-H | C_16_H_18_N_2_O_6_ | 334.32 |
| 36 | 4-O-β-Galactopyranosyl-D-mannopyranose | 387.1134 | 0.6 | M-H, M+FA-H | C_12_H_22_O_11_ | 342.30 |
| 37 | Chrysoeriol | 299.0557 | 8.23 | M-H | C_16_H_12_O_6_ | 300.263 |
| 38 | Narcissin | 623.1613 | 5.83 | M-H | C_28_H_32_O_16_ | 624.54 |
| 39 | D-(+)-Raffinose | 549.1664 | 0.82 | M+FA-H | C_18_H_32_O_16_ | 504.44 |
| 40 | Kaempferol 3-O-rutinoside | 593.1506 | 5.72 | M-H | C_27_H_30_O_15_ | 594.52 |
| 41 | 1-Palmitoyl-2-hydroxy-sn-glycero-3-phospho-(1'-rac-glycerol) | 483.2711 | 13.03 | M-H | C_22_H_45_O_9_P | 506.54 |
| 42 | Glucobrassicin | 447.0527 | 2.56 | M-H | C_16_H_20_N_2_O_9_S_2_ | 448.47 |
| 43 | Astragalin | 447.0922 | 5.88 | M-H | C_21_H_20_O_11_ | 448.38 |


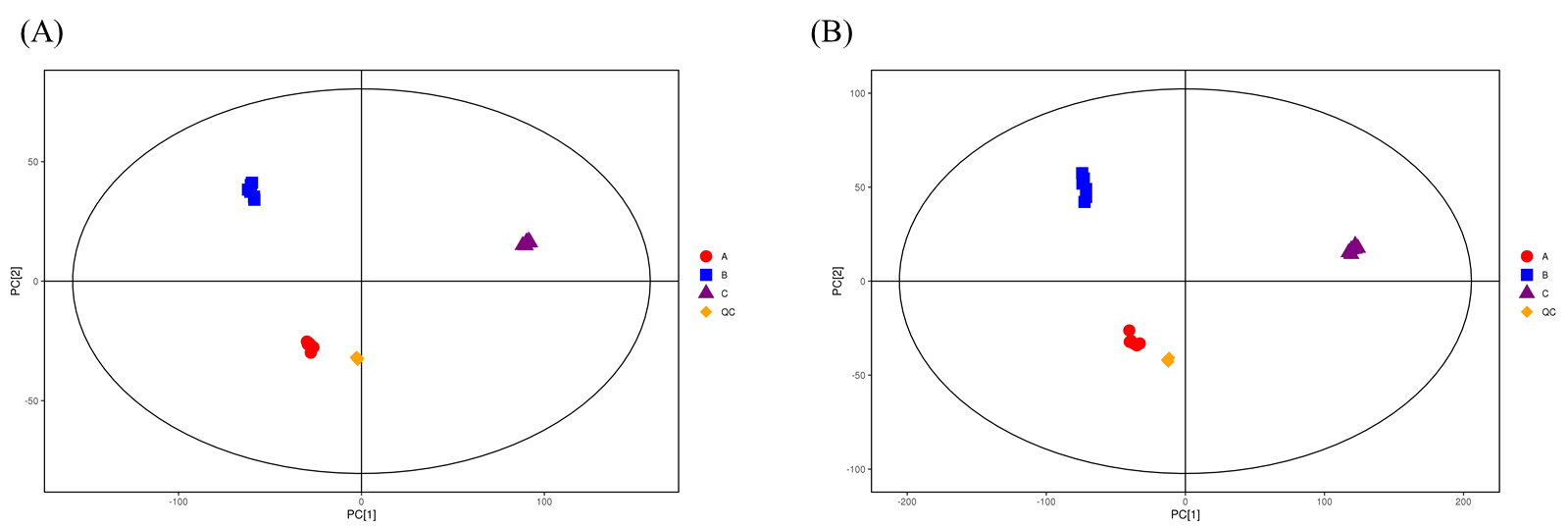


Figure S1. Score scatter plot for PCA model total sample with QC. The positive ion mode was on the left, the negative ion mode was on the right.


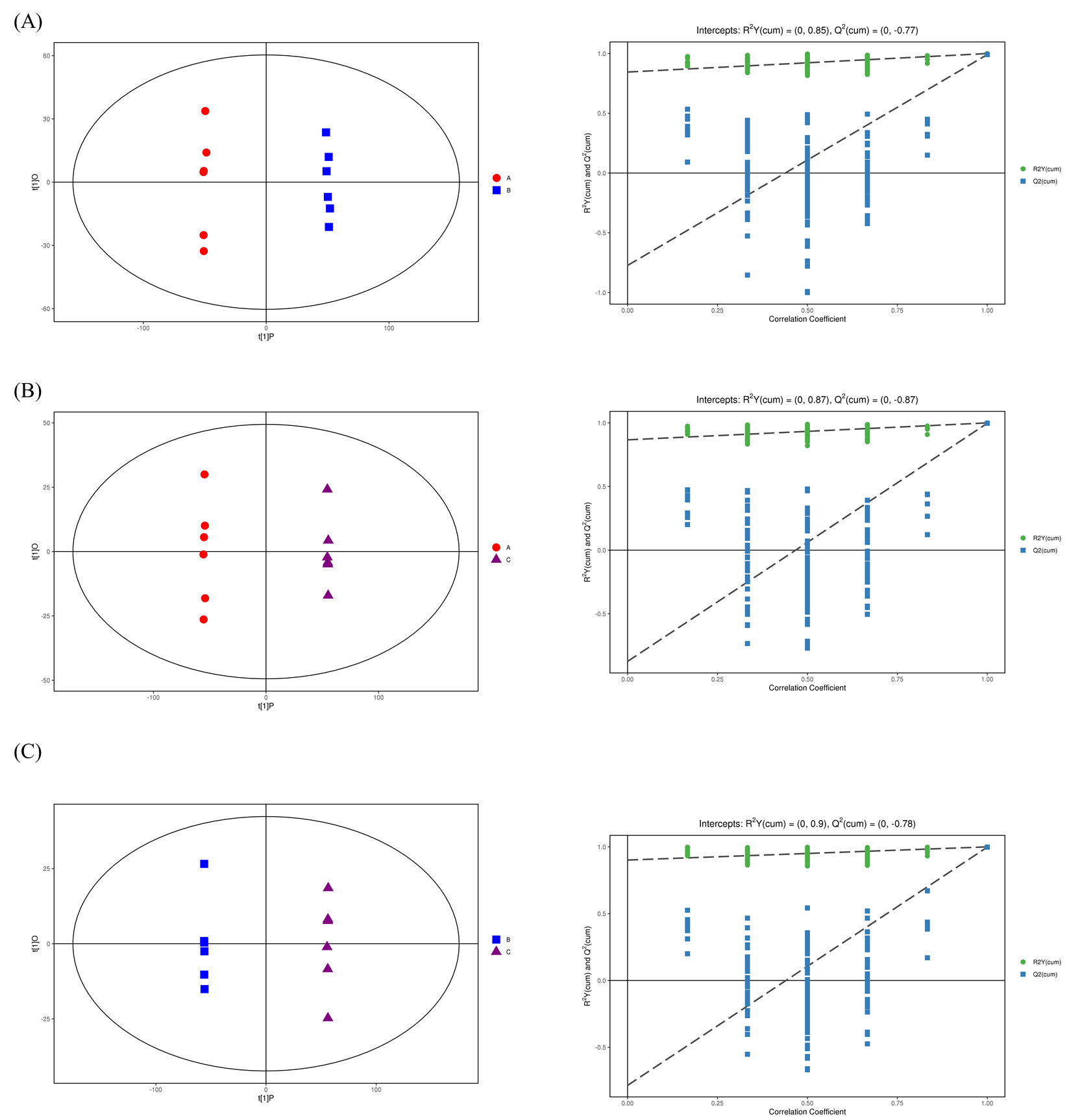


Figure S2. Score scatter plot and Permutation test of OPLS-DA model. (A) group A vs B. (B) group A vs C. (C) group B vs C.

Table S5. The differential metabolites

|  |  | **In positive ion method** | **In negative ion method** |
| --- | --- | --- | --- |
| **Common compounds** | **A-B vs A-C** | Coniferyl aldehyde | Spionoside B |
|  |  | N-Oleoylglycine |  |
|  | **A-B vs B-C** | L-Arginine | 9,10-Dihydroxy-12Z-octadecenoic acid |
|  |  | 9-Oxo-11-(3-pentyl-2-oxiranyl)-10E-undecenoic acid | Guanosine |
|  |  | 9(10)-Epoxy-12Z-octadecenoic acid | 3,4-Dihydroxybenzaldehyde |
|  |  | Vanillic acid | 6-Methoxyindoline-2,3-dione |
|  | **A-C vs B-C** | Methyl-γ-linolenate | Cappariloside A |
|  |  | Diosmetin | Chrysoeriol |
|  |  | Kaempferol 3-O-rutinoside | Kaempferol 3-O-rutinoside |
|  |  | Linoleoyl ethanolamide | 5-Hydroxyindole-3-acetic acid |
|  |  | Alangionoside C | Corchoionoside C |
|  |  | (+)-Abscisic acid | Citric acid |
|  |  | N-Oleoylethanolamine | Glucobrassicin |
|  |  | Ricinoleic acid methyl ester | 4-O-β-Galactopyranosyl-D-mannopyranose |
|  |  | Corchoionoside C | Narcissin |
|  |  | Palmitoyl ethanolamide | Astragalin |
|  |  |  | L-(-)-3-Phenyllactic acid |
| **Unique compounds** | **A vs B** |  | DL-Malic acid |
|  |  |  | 1-Palmitoyl-2-hydroxy-sn-glycero-3-phosphoethanolamine |
|  |  |  | D-(+)-Raffinose |
|  | **A vs C** | Quinolin-5-ol | Methyl hexadecanoate |
|  |  | 13-Keto-9Z,11E-octadecadienoic acid | Dodecanedioic acid |
|  |  |  | 3-O-β-D-Glucopyranosyl sitosterol |
|  | **B vs C** | Monolinolenin (9c,12c,15c) |  |
|  |  | (Z)-6-Octadecenoic acid |  |
|  |  | Stachydrine |  |


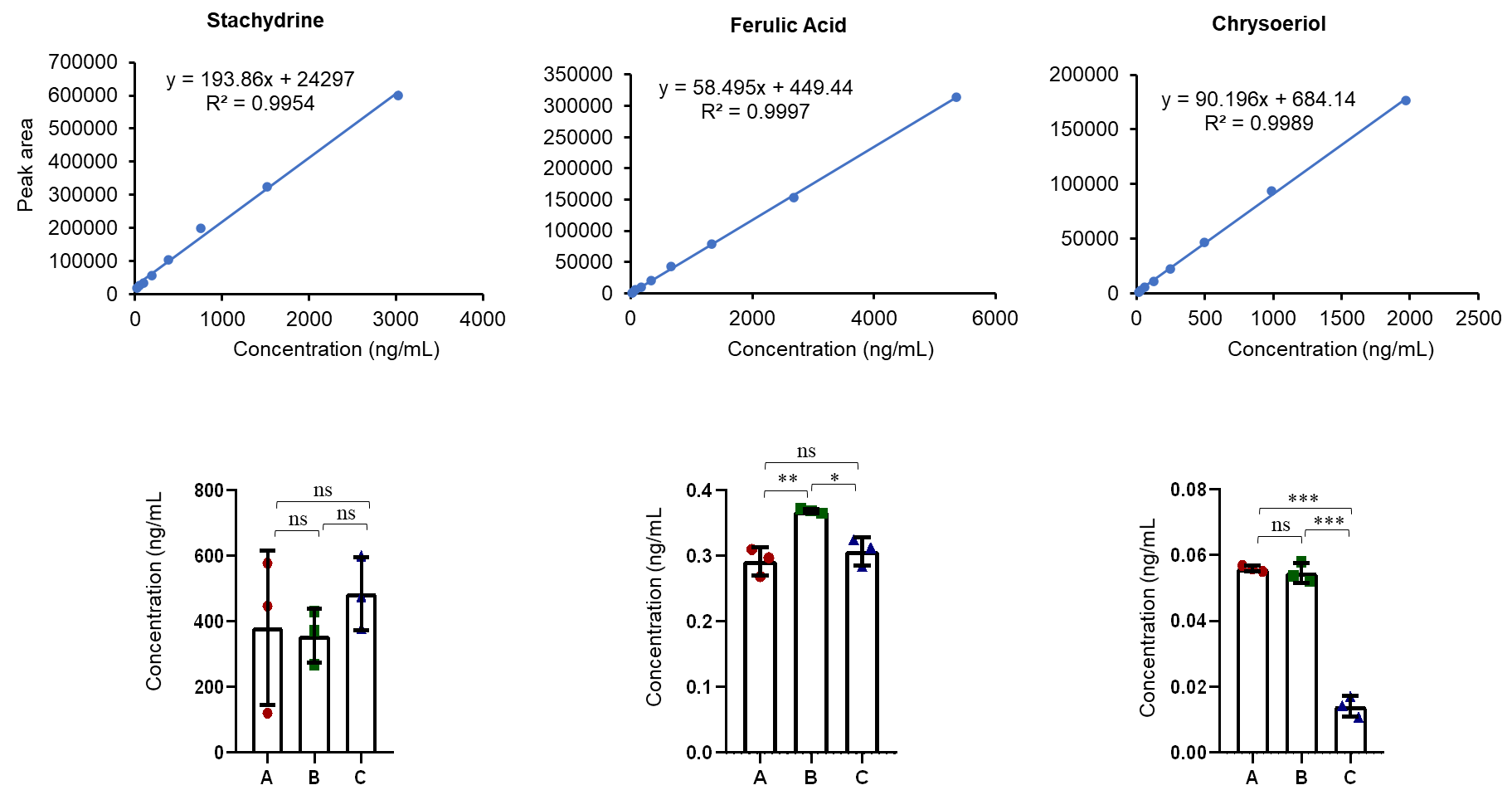


Figure S3. Validation of metabolite. Data are means ±SE (n = 3).

ns: no significance; ^*^*P* < 0.05, ^**^*P* < 0.01, ^***^*P* < 0.001


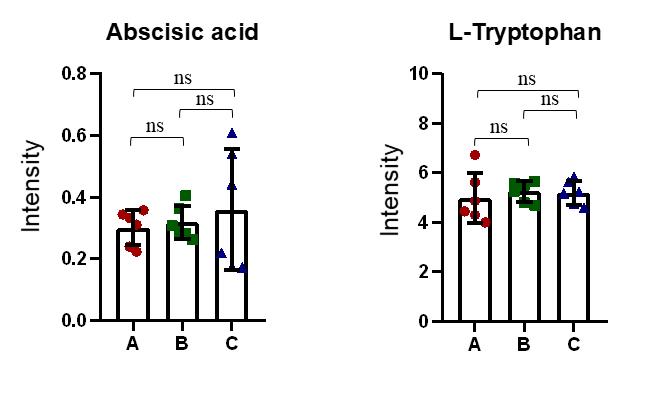


Figure S4. Contents of abscisic acid and tryptophan.

ns: no significance


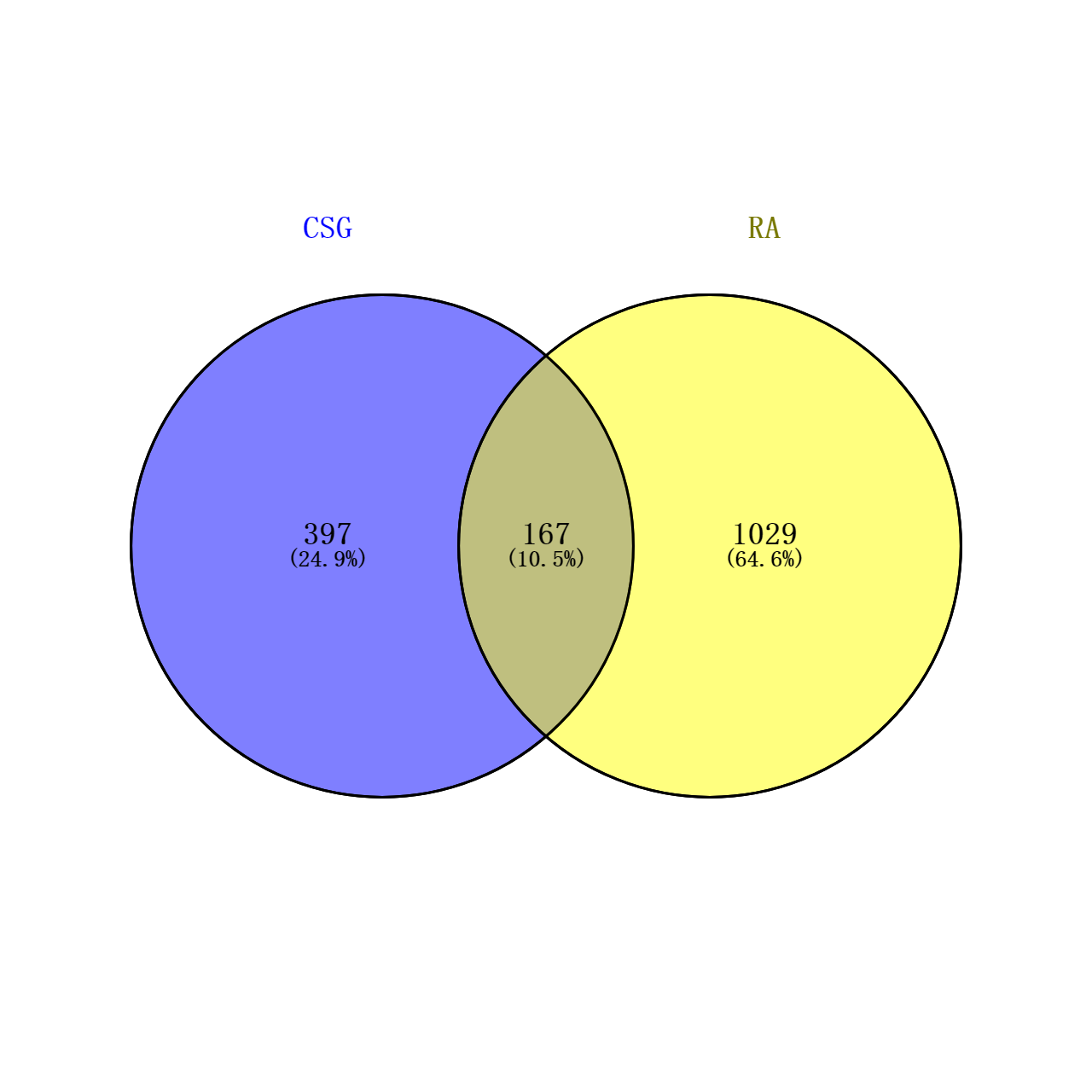


Figure S5. Venn diagram for intersection analysis between compound targets and RA targets.


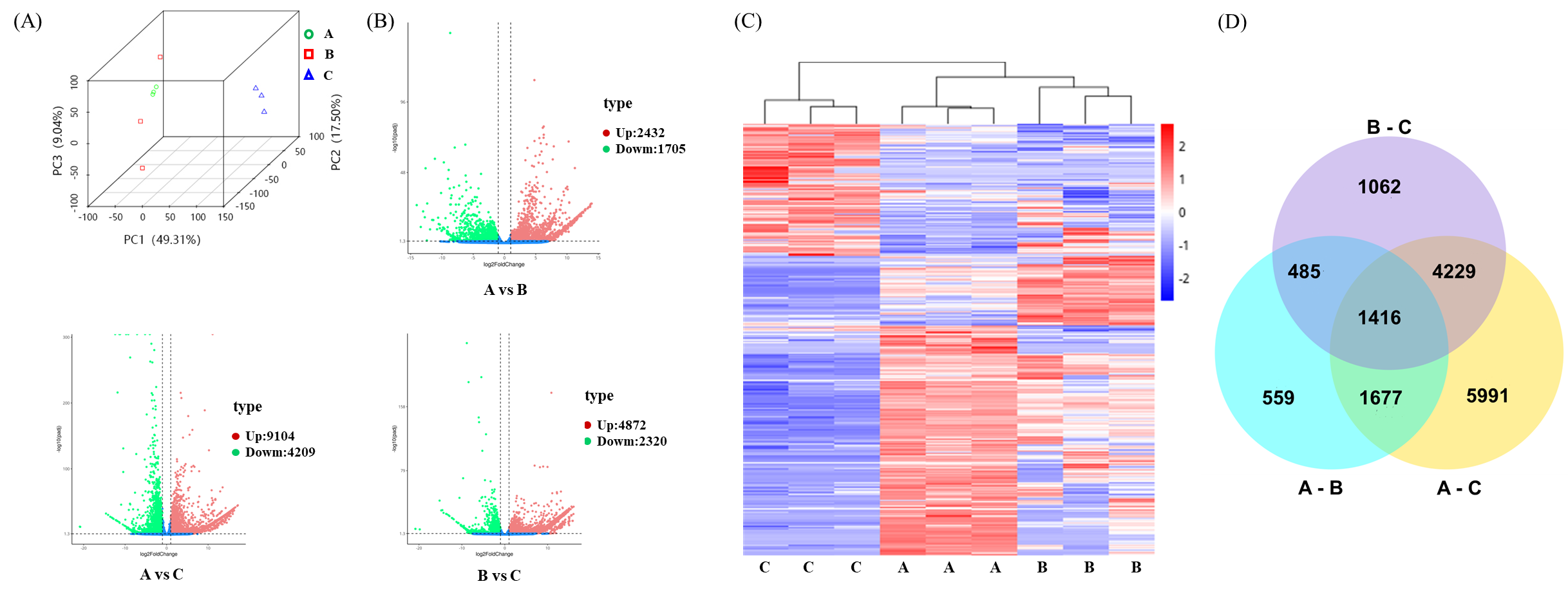


Figure S6. Transcriptomic analysis of C. spinosa fruits. (A) PCA score map. (B) Volcano map. (C) HCA map. (D) Venn map. (n = 3).


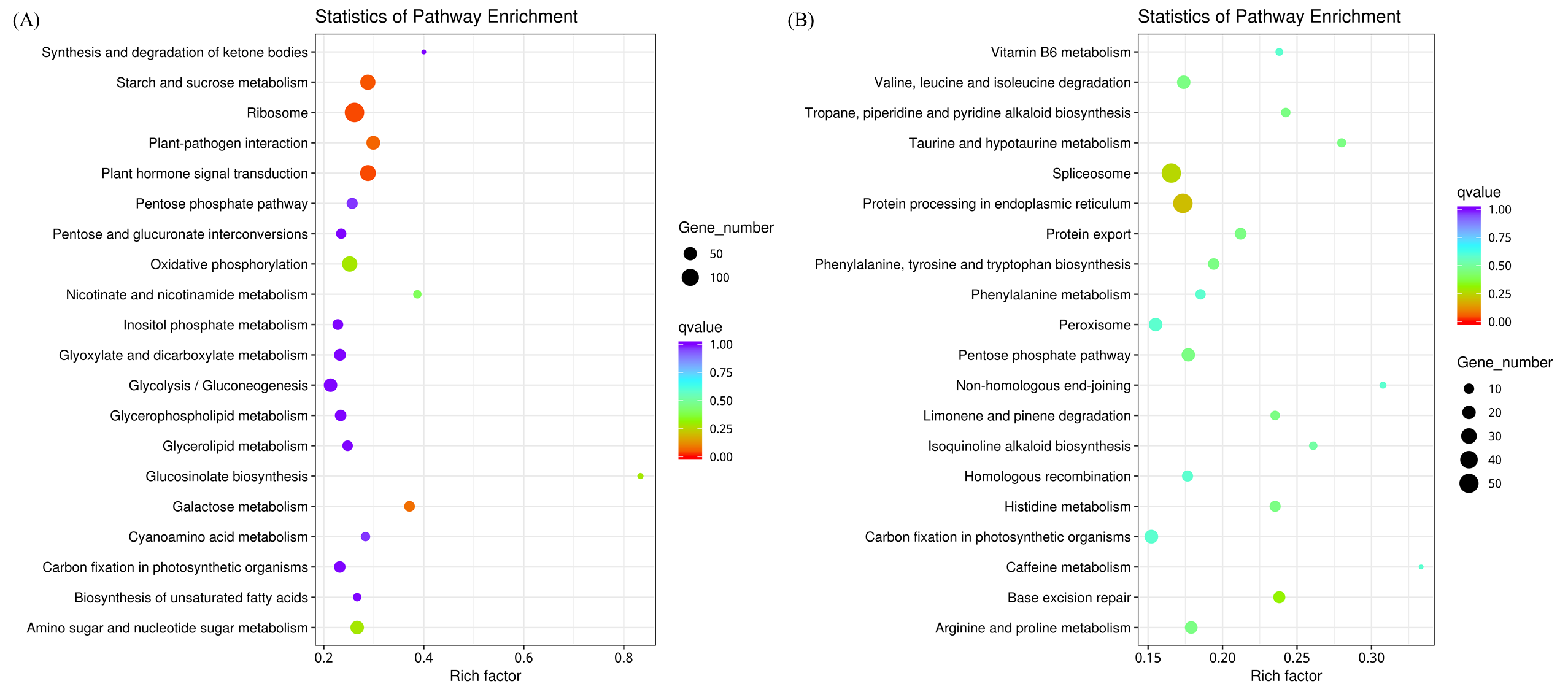


Figure S7. KEGG pathway enrichment (A vs C). (A) up-regulated pathway. (B)down-regulated pathway was on the right.

Table S1. The Primer sequences for qRT‑PCR

| Gene id | | F | R | |
| --- | --- | --- | --- | --- |
| 2209 | GCTTCGTAGCAGTTCTTCAGGAATTTC | | GGCGTTGAGCATGTTGGAGGAG | |
| 2180 | CTCTTCCGCAGAACAATTCCTCCTC | | | CTTCACCAACAAGGTAGTCCAGCAG |
| 4290 | TTGCGGCGACGTTGATGTTCTC | | | GAGGAAGAATATAGCGAGCACCAAGG |
| 2355 | CGGTTACATCGCCTCTTGGATCG | | | GTCAATCTCTCCTTCGCTCCTTCG |
| 7764 | ATTACCAGAGATACGAACACGAGAACC | | | GCAGGAATACGAGAACGGCAAGG |
| 3246 | CTTTGGCAATGGCGTCTGTTGAAG | | | CAACCGAGTGCTTGACCTGATAGAG |


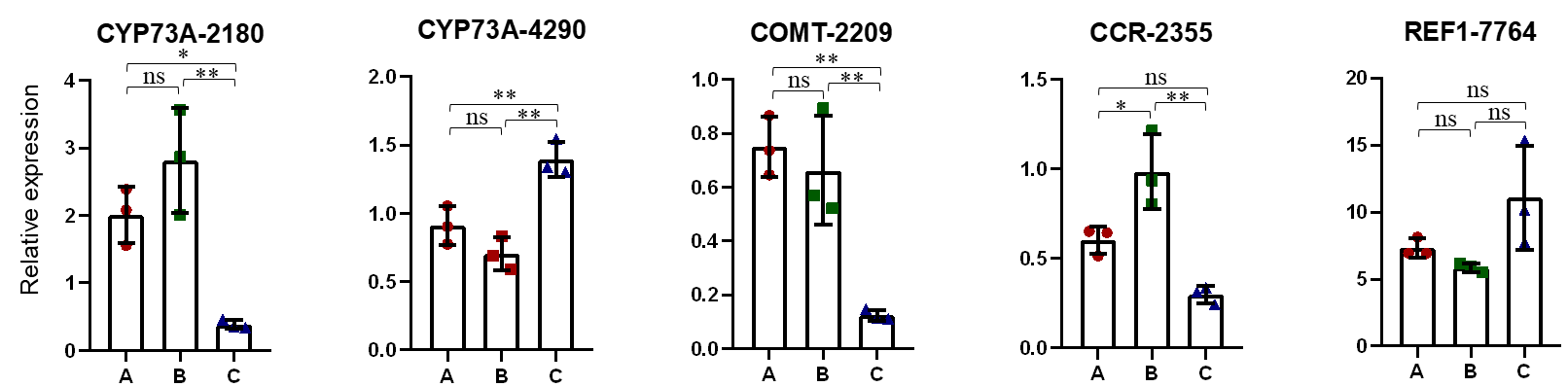


Figure S8. Validation of genes expression by qRT‑PCR. Data are means ±SE (n = 3).

ns: no significance; ^*^*P* < 0.05, ^**^*P* < 0.01, ^***^*P* < 0.001.
